# Complicated appendicular diverticulitis

**DOI:** 10.1002/jmrs.573

**Published:** 2022-02-23

**Authors:** Zachary J. Drew, Sriya Chakrabarty, Raghvendra Malghan

**Affiliations:** ^1^ Department of Medical Imaging Royal Brisbane and Women’s Hospital Queensland Australia; ^2^ Department of Medical Imaging Townsville University Hospital Townsville Queensland Australia; ^3^ Senior Staff Specialist Department of Medical Imaging Townsville University Hospital Townsville Queensland Australia

**Keywords:** Appendiceal diverticulitis, appendiceal diverticulosis, appendicitis, appendicular diverticulitis, diverticulitis

## Abstract

Appendiceal diverticulitis, a frequently underdiagnosed entity, differs from typical appendicitis by the presence of an inflamed appendiceal diverticulum. Appendiceal diverticulitis is a surgical emergency which has an increased risk of perforation compared to typical appendicitis. We will discuss a surgically and pathologically confirmed case of complicated appendiceal diverticulitis and its management implications.

## Graphical abstract

Appendiceal diverticulitis, a frequently underdiagnosed entity, differs from typical appendicitis by the presence of an inflamed appendiceal diverticulum. Appendiceal diverticulitis is a surgical emergency which has an increased risk of perforation compared to typical appendicitis. We discuss a surgically and pathologically confirmed case of complicated appendiceal diverticulitis and its management implications.

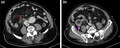

## Introduction

Acute appendicitis is one of the most common surgical causes of acute abdominal pain. Appendicular diverticulitis is a rare but important counterpart to appendicitis.^1^

Acquired bowel diverticula are blind‐ended outpouchings arising through muscular mural defects connecting with the bowel lumen.^2^ Radiological diagnosis of appendiceal diverticulitis is based on the identification of a diverticula arising from the appendix, but can be difficult in the setting of advanced inflammatory changes or perforation which can obscure the presence of diverticula.^3^ Its incidence may be underestimated due to lack of awareness of its subtle radiological findings and clinico‐radiological overlap with appendicitis.^3^, ^4^ The resultant delayed diagnosis is associated with significant complications including a sixfold increase in the risk of perforation.^1^, ^5^

## Case Report

Informed consent was obtained from the patient for publication of this case report.

A 75‐year‐old male presented to emergency with 2 days of peri‐umbilical and right iliac fossa pain, with tenderness at McBurney’s point and a raised white cell count (15.2x10^9/L).

**Figure 1:**
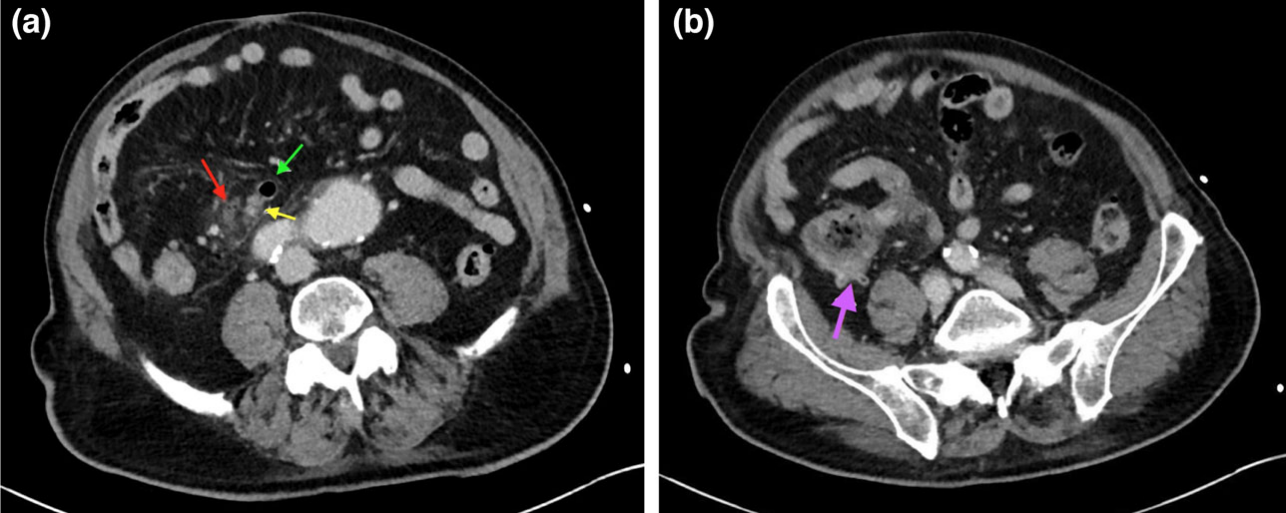
(A) Axial slice demonstrating the thickened, enhancing appendix (yellow arrow) arising from the caecum, with a 15mm gas containing diverticulum at the appendix tip (green arrow) with adjacent fat stranding / free fluid and gas (red arrow). (B) The patient has moderate diverticulosis throughout the large bowel including the right colon (purple arrow).

**Figure 2:**
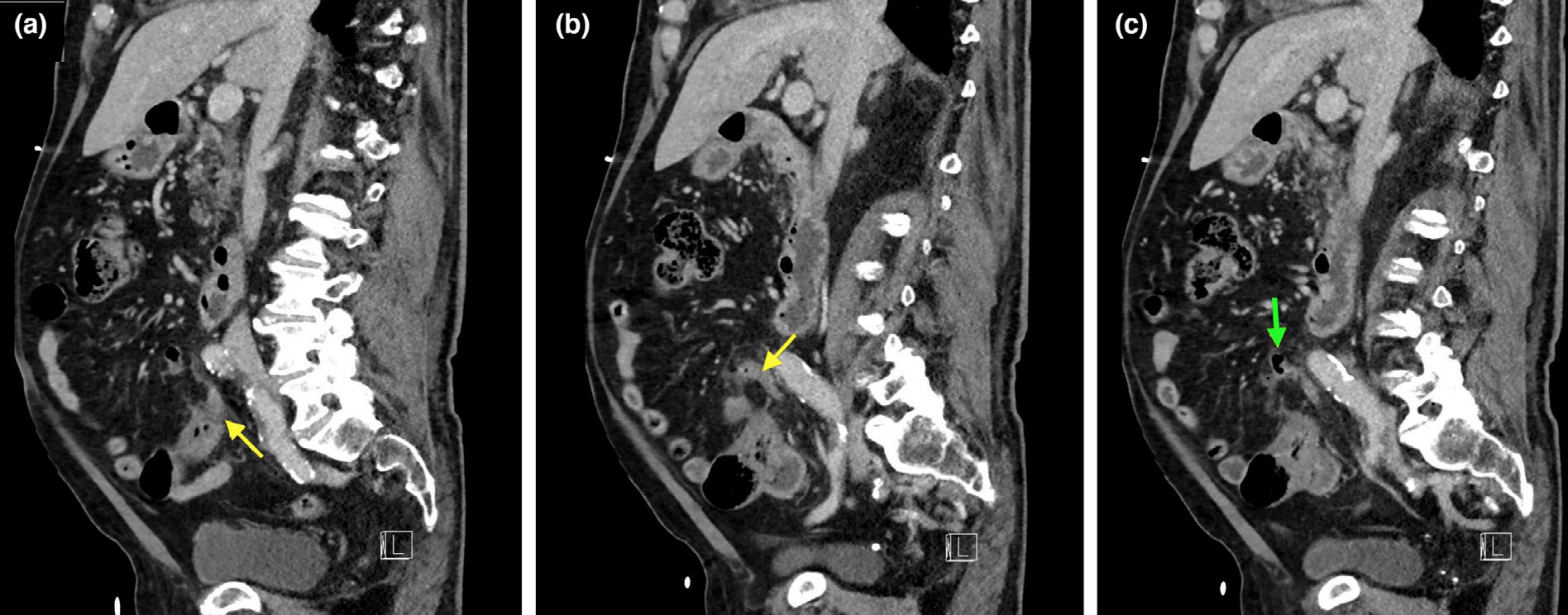
Serial sagittal slices of the appendiceal diverticulitis. (A, B) Appendix arising from the caecum (yellow arrow). (C) Appendiceal diverticulum (green arrow) arising from the superior aspect of the appendix, with adjacent inflammatory changes evident.

A computed tomography (CT) revealed a thin‐walled 15‐mm diverticulum arising from the appendix tip (Figs 1 and 2), with adjacent stranding and a gas containing fluid collection indicating perforation. The appendix was dilated (13mm), with thickened, hyper‐enhancing walls.

**Figure 3:**
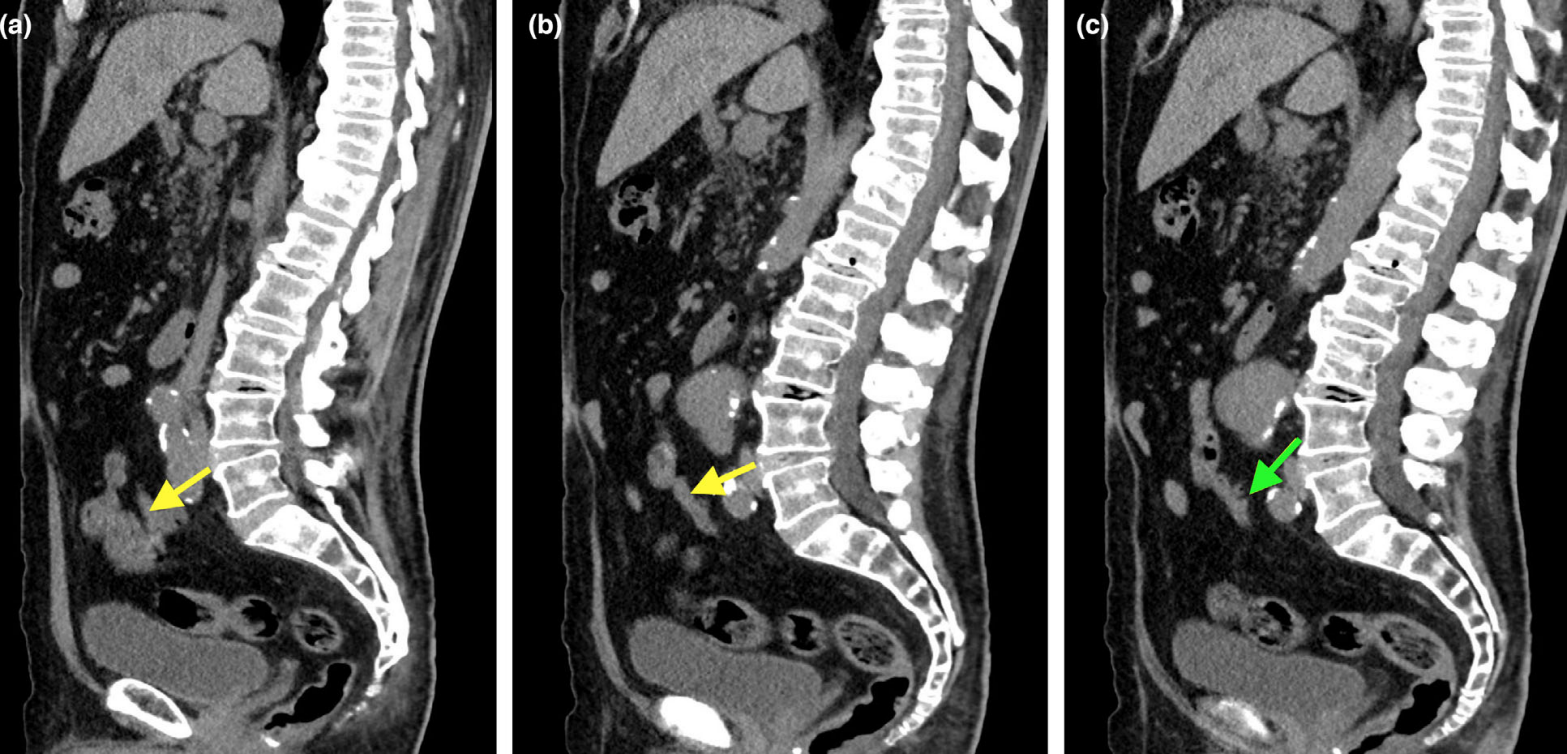
Serial non‐contrast sagittal CT appearance of the patient’s appendix (yellow arrow in A, B) 2 years prior, with small diverticula (green arrow in C), with no features of active inflammation.

CT imaging 2 years prior (Fig. 3) revealed a normal calibre appendix with incidental three small, non‐inflamed diverticula.

The patient proceeded to appendicectomy, demonstrating appendiceal rupture with gangrenous change.

**Figure 4:**
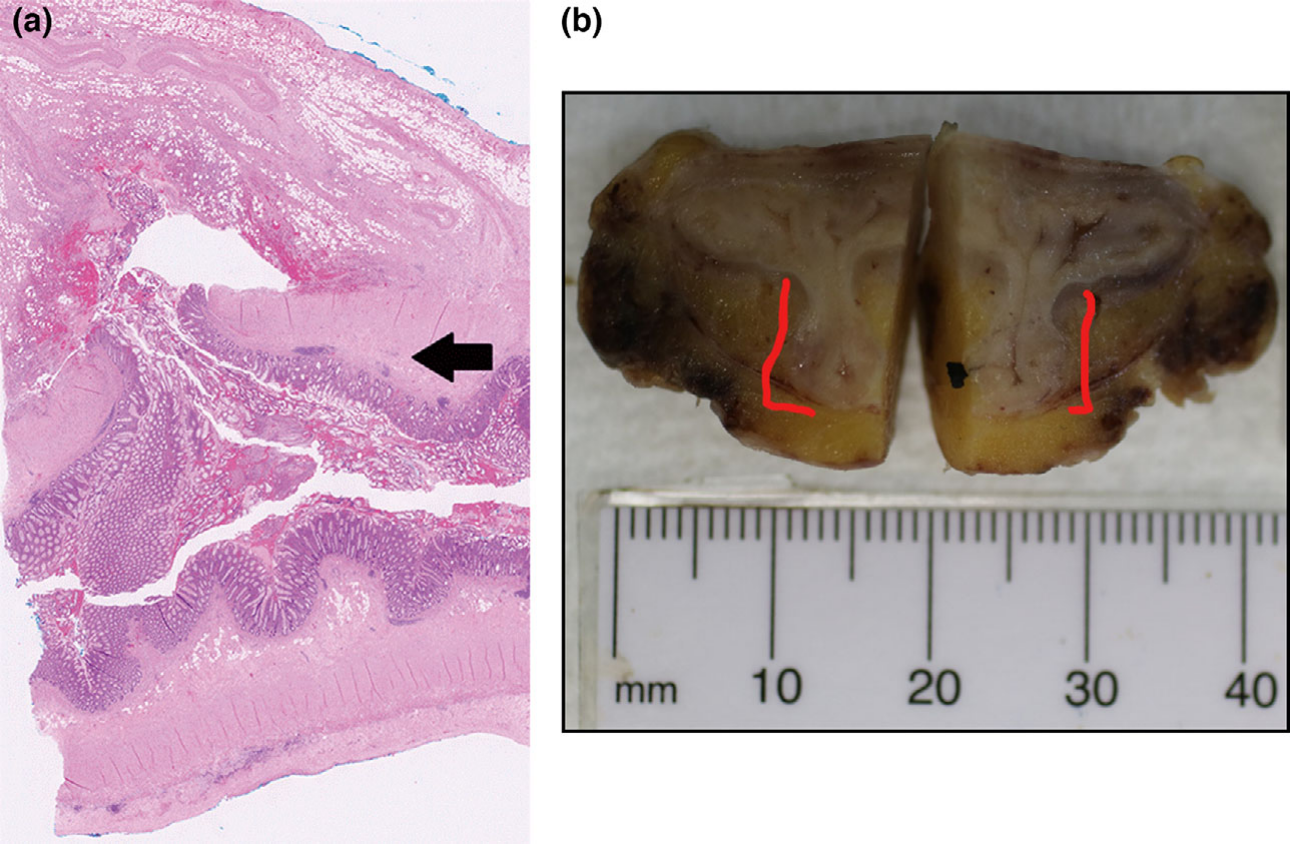
(A) Microscopic and (B) macroscopic images of the specimen. (A) Microscopic appearance of the diverticulum (diverticulum labelled with black arrow). (B) Red markers demarcate the macroscopic false diverticulum. Section has been bisected.

Pathology confirmed that the appendix contained a perforated diverticulum at its tip (Fig. 4). Microscopically, there was extensive mucosal ulceration, transmural inflammation and wall necrosis.

## Discussion

Appendiceal diverticulitis is rare, with reported surgical incidence up to 3.7%.^1^ A higher incidence of 9.7% observed in the series by Ito et al. was attributed to careful resection and pathological processing of the specimens.^4^

In practice, preoperative radiological diagnosis is also frequently missed.^2^, ^3^ A contributing factor is likely under‐recognition of the condition and its significance. Another factor may be the potential subtlety of its findings.^2^, ^3^ The key imaging differentiation is the identification of a diverticula arising from the appendix at the epicentre of the inflammatory change. Due to the small size of the diverticula and potential obscuration from adjacent stranding, identification may require a more deliberate search pattern and interrogation of multiple‐planes of imaging than would otherwise be typical.^2^, ^3^

Radiologically, CT is the imaging modality of choice, by which the patterns of appendicular diverticular disease can be recognised.^5^ In our case, there is CT evidence of progression from a non‐inflammatory to inflammatory state involving the diverticula and the appendix. The presence of advanced necrosis within the appendix would have clinically masked any potential differentiation of acute appendicitis from diverticular symptoms.

The rate of perforation in appendiceal diverticulitis ranges from 30 to 70%, more than four times higher than appendicitis, which is likely due to the thin‐walled diverticulum providing a weak point for rupture, further necessitating distinction between the two.^1^, ^2^, ^5^

In regard to management, there is growing evidence and support for early appendicectomy for appendiceal diverticulitis and also for diverticulosis.^2^, ^5^, ^6^, ^7^, ^8^, ^9^ In Osada et al.'s case series of seven patients with appendiceal diverticulitis, each of the two cases which were initially managed conservatively subsequently demonstrated perforation, eventually requiring surgery.^5^ Some of the recent literature even discuss a role for prophylactic appendicectomy when appendicular diverticula are found incidentally during abdominal surgery because of the high risk of future diverticulitis leading to perforation as well as an increased association with neoplasms.^2^, ^6^, ^7^, ^8^, ^9^ This potential significant impact on management highlights the importance of radiological diagnosis.

Imaging with modern thin slice CT scanners, awareness of the subtle findings of this entity and comparative studies, (as in our case), will help in detecting appendiceal diverticula and aid in differentiating diverticulitis from isolated or accompanying appendicitis.

Lee et al. postulate that accompanying appendicitis is often secondary and that these two subtypes (i.e. diverticulitis with or without appendicitis) represent different stages in the progression of same disease process.^3^ Appendiceal diverticulitis is postulated to arise from primary inflammation in appendiceal diverticula, which can then lead to reactive changes in the adjacent appendix subserosa / serosa and peri‐appendiceal space.^3^

In summary, significant complications and morbidity associated with appendicular diverticular disease could be potentially avoided by early diagnosis and management as suggested by our case.
